# (*E*)-*N*′-(4-Pyridylmethyl­ene)-4-(8-quinol­yl­oxy)butanohydrazide 0.25-hydrate

**DOI:** 10.1107/S160053680900110X

**Published:** 2009-01-14

**Authors:** Min-E Chen, Jia-Ming Li

**Affiliations:** a Department of Chemistry and Biology, Laboratory of Beibu Gulf Marine Protection and Exploitation, Qinzhou University, Qinzhou, Guangxi 535000, People’s Republic of China

## Abstract

The asymmetric unit of the title compound, C_19_H_18_N_4_O_2_·0.25H_2_O, contains two organic mol­ecules and a solvent water mol­ecule with 50% occupancy. The two molecules differ in their conformations: in one mol­ecule it is (+)*gauche*-*trans*-*trans*-(+)*gauche*-*trans*, whereas in the other it is (−)*gauche*-*trans*-*trans*-(−)*gauche*-*trans*. The dihedral angles between the pyridine ring and the quinoline ring system are 67.4 (3) and 68.0 (2)°. Mol­ecules are linked into a supra­molecular two-dimensional array *via* N—H⋯N hydrogen bonds, with each partially occupied water mol­ecule connected *via* an O—H⋯O hydrogen bond. C—H⋯O inter­actions are also present.

## Related literature

For general background, see: Cai *et al.* (2003 ▶); Chen *et al.* (2005 ▶); Park *et al.* (2006 ▶); Karmakar *et al.* (2007 ▶). For related structures, see: Zheng *et al.* (2006 ▶, 2007 ▶, 2008 ▶); Xie *et al.* (2008 ▶).
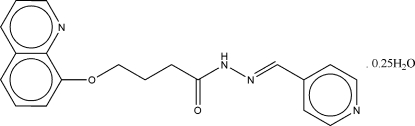


## Experimental

### 

#### Crystal data

C_19_H_18_N_4_O_2_·0.25H_2_O
*M* *_r_* = 1355.51
Monoclinic, 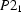

*a* = 8.8816 (13) Å
*b* = 17.420 (2) Å
*c* = 11.3624 (17) Å
β = 100.765 (3)°
*V* = 1727.0 (4) Å^3^
*Z* = 1
Mo *K*α radiation
μ = 0.09 mm^−1^
*T* = 295 K
0.32 × 0.26 × 0.22 mm

#### Data collection

Bruker SMART CCD area-detector diffractometer
Absorption correction: multi-scan (*SADABS*; Sheldrick, 1996 ▶) *T*_min_ = 0.972, *T*_max_ = 0.981
9142 measured reflections
3169 independent reflections
1927 reflections with *I* > 2σ(*I*)
*R*_int_ = 0.050

#### Refinement

*R*[*F* ^2^ > 2σ(*F* ^2^)] = 0.058
*wR*(*F* ^2^) = 0.162
*S* = 1.01
3169 reflections
460 parameters
H-atom parameters constrained
Δρ_max_ = 0.32 e Å^−3^
Δρ_min_ = −0.21 e Å^−3^

### 

Data collection: *SMART* (Bruker, 2007 ▶); cell refinement: *SAINT* (Bruker, 2007 ▶); data reduction: *SAINT*; program(s) used to solve structure: *SHELXS97* (Sheldrick, 2008 ▶); program(s) used to refine structure: *SHELXL97* (Sheldrick, 2008 ▶); molecular graphics: *SHELXTL* (Sheldrick, 2008 ▶); software used to prepare material for publication: *SHELXTL*.

## Supplementary Material

Crystal structure: contains datablocks global, I. DOI: 10.1107/S160053680900110X/tk2355sup1.cif

Structure factors: contains datablocks I. DOI: 10.1107/S160053680900110X/tk2355Isup2.hkl

Additional supplementary materials:  crystallographic information; 3D view; checkCIF report

## Figures and Tables

**Table 1:** Hydrogen-bond geometry (Å, °)

*D*—H⋯*A*	*D*—H	H⋯*A*	*D*⋯*A*	*D*—H⋯*A*
C22—H22⋯O4^i^	0.93	2.51	3.224 (7)	134
C7—H7⋯N8^ii^	0.93	2.55	3.466 (9)	167
C3—H3⋯O2^iii^	0.93	2.52	3.353 (7)	150
C2—H1⋯N4^iv^	0.93	2.55	3.390 (9)	150
O5—H39⋯O4^v^	0.85	2.17	2.964 (10)	156
N6—H6⋯N1^iii^	0.86	2.10	2.934 (6)	163
N2—H2⋯N5^i^	0.86	2.25	3.077 (7)	161

Symmetry codes: (i) 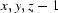; (ii) 
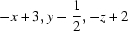
; (iii) 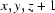; (iv) 
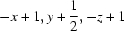
; (v) 
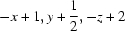
.

**Fig. 1.**
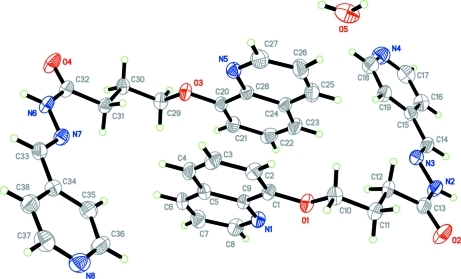
The molecular structures of the two independent molecules and the water molecule of crystallization (50% site occupancy) in (I), showing atom numbering scheme and displacement ellipsoids at the 30% probability level.

## supplementary crystallographic information

### Comment

The coordination chemistry of 8-hydroxyquinoline and its derivatives is well studied (Cai *et al.*, 2003; Chen *et al.*, 2005; Park *et al.*, 2006; Karmakar *et al.* 2007). In the course of our studies searching for good extractants of metal ions, the title compound, (I), was synthesized and its crystal structure determined. The asymmetric unit comprises two independent molecules which differ in conformation and a water molecule with a 50% site occupancy factor. In one molecule, the conformation along the O1—C10—C11—C12—C13—N2—N3—C14 bond sequence is (+)*gauche*-*trans*-*trans*-(+)*gauche*-*trans*, whereas in the second molecule the conformation is (-)*gauche*-*trans*-*trans*-(-)*gauche*-*trans* along the corresponding O3—C29—C30—C31—C32—N6—N7—C33 bond sequence. Despite these differences, the dihedral angles between the mean planes of the pyridine and quinoline rings are not very different, i.e. 67.4 (3)° and 68.0 (2)° for the molecules containing atoms O1 and O3, respectively. The two independent molecules are linked to a supermolecular 2D array *via* N—H···N hydrogen bonds supported by C—H···O contacts, Table 1. Each partially occupied water molecules is connected to a layer via a O—H···O hydrogen bond.

### Experimental

4-(Quinolin-8-yloxy)butanohydrazide (0.01 mol), 4-pyridylaldehyde (0.01 mol), ethanol (40 ml) and some drops of acetic acid were added to a 100 ml flask, and refluxed for 8 h. After cooling to room temperature, the mixture was filtered. Colourless crystals were obtained by slow evaporation of a tetrahydrofuran solution over a period of 2 days; m.p. 438 K. Analysis found: C 67.34, H 5.50, N 16.53; C_76_H_74_N_16_O_9_ requires: C 67.30, H 5.54, N 16.51.

### Refinement

All H atoms were placed in idealized positions (C—H = 0.93–0.97 Å, N—H = 0.86 Å and O—H = 0.85 Å) and refined in the riding model approximation with *U*_iso_(H) = 1.2*U*_eq_(C or N) and *U*_iso_(H) = 1.5*U*_eq_(O). The water molecule was included in the model with a 50% site occupancy factor based on elemental analysis and refinement. In the absence of significant anomalous scattering effects, X Friedel pairs were averaged in the final refinement.

### Crystal data

**Table:**

C_19_H_18_N_4_O_2_·0.25H_2_O	*F*(000) = 714
*M**_r_* = 1355.51	*D*_x_ = 1.303 Mg m^−^^3^
Monoclinic, *P*2_1_	Melting point: 438 K
Hall symbol: P 2yb	Mo *K*α radiation, λ = 0.71073 Å
*a* = 8.8816 (13) Å	Cell parameters from 1165 reflections
*b* = 17.420 (2) Å	θ = 2.2–20.0°
*c* = 11.3624 (17) Å	µ = 0.09 mm^−^^1^
β = 100.765 (3)°	*T* = 295 K
*V* = 1727.0 (4) Å^3^	Block, colorless
*Z* = 1	0.32 × 0.26 × 0.22 mm

### Data collection

**Table:**

Bruker SMART CCD area-detector diffractometer	3169 independent reflections
Radiation source: fine-focus sealed tube	1927 reflections with *I* > 2σ(*I*)
graphite	*R*_int_ = 0.050
φ and ω scans	θ_max_ = 25.1°, θ_min_ = 1.8°
Absorption correction: multi-scan (*SADABS*; Sheldrick, 1996)	*h* = −10→10
*T*_min_ = 0.972, *T*_max_ = 0.981	*k* = −13→20
9142 measured reflections	*l* = −13→13

### Refinement

**Table:**

Refinement on *F*^2^	Secondary atom site location: difference Fourier map
Least-squares matrix: full	Hydrogen site location: inferred from neighbouring sites
*R*[*F*^2^ > 2σ(*F*^2^)] = 0.058	H-atom parameters constrained
*wR*(*F*^2^) = 0.162	*w* = 1/[σ^2^(*F*_o_^2^) + (0.0877*P*)^2^ + 0.001*P*] where *P* = (*F*_o_^2^ + 2*F*_c_^2^)/3
*S* = 1.01	(Δ/σ)_max_ < 0.001
3169 reflections	Δρ_max_ = 0.32 e Å^−^^3^
460 parameters	Δρ_min_ = −0.21 e Å^−^^3^
0 restraints	Absolute structure: nd
Primary atom site location: structure-invariant direct methods	

### Special details

**Table:**

Geometry. All e.s.d.'s (except the e.s.d. in the dihedral angle between two l.s. planes) are estimated using the full covariance matrix. The cell e.s.d.'s are taken into account individually in the estimation of e.s.d.'s in distances, angles and torsion angles; correlations between e.s.d.'s in cell parameters are only used when they are defined by crystal symmetry. An approximate (isotropic) treatment of cell e.s.d.'s is used for estimating e.s.d.'s involving l.s. planes.
Refinement. Refinement of *F*^2^ against ALL reflections. The weighted *R*-factor *wR* and goodness of fit *S* are based on *F*^2^, conventional *R*-factors *R* are based on *F*, with *F* set to zero for negative *F*^2^. The threshold expression of *F*^2^ > σ(*F*^2^) is used only for calculating *R*-factors(gt) *etc*. and is not relevant to the choice of reflections for refinement. *R*-factors based on *F*^2^ are statistically about twice as large as those based on *F*, and *R*- factors based on ALL data will be even larger.

### Fractional atomic coordinates and isotropic or equivalent isotropic displacement parameters (Å^2^)

**Table:**

	*x*	*y*	*z*	*U*_iso_*/*U*_eq_	Occ. (<1)
N1	1.1571 (5)	0.0923 (3)	0.6124 (4)	0.0478 (12)	
N2	0.7191 (6)	0.1522 (3)	0.1045 (4)	0.0566 (14)	
H2	0.7211	0.1460	0.0297	0.068*	
N3	0.6437 (6)	0.1008 (3)	0.1636 (4)	0.0510 (13)	
N4	0.3307 (7)	−0.1253 (4)	0.2546 (6)	0.0817 (18)	
N5	0.6503 (5)	0.1139 (3)	0.8355 (4)	0.0495 (12)	
N6	1.0930 (6)	0.0735 (3)	1.3512 (4)	0.0510 (13)	
H6	1.0909	0.0810	1.4257	0.061*	
N7	1.1691 (6)	0.1246 (3)	1.2916 (4)	0.0474 (12)	
N8	1.4779 (7)	0.3522 (3)	1.1941 (6)	0.0674 (15)	
O1	0.9800 (4)	0.2139 (2)	0.5363 (3)	0.0497 (10)	
O2	0.8522 (6)	0.2626 (3)	0.1125 (4)	0.0769 (14)	
O3	0.8302 (5)	−0.0034 (2)	0.9236 (3)	0.0511 (10)	
O4	0.9623 (6)	−0.0348 (2)	1.3516 (4)	0.0707 (14)	
O5	0.2645 (9)	0.3398 (5)	0.6539 (8)	0.072 (3)	0.50
H39	0.2124	0.3805	0.6357	0.107*	0.50
H40	0.2794	0.3362	0.7297	0.107*	0.50
C1	1.0067 (7)	0.1999 (3)	0.6560 (5)	0.0430 (14)	
C2	0.9470 (7)	0.2422 (3)	0.7384 (5)	0.0491 (15)	
H1	0.8840	0.2840	0.7134	0.059*	
C3	0.9807 (7)	0.2225 (4)	0.8599 (5)	0.0557 (16)	
H3	0.9389	0.2514	0.9147	0.067*	
C4	1.0719 (7)	0.1628 (4)	0.8992 (5)	0.0569 (17)	
H4	1.0932	0.1515	0.9806	0.068*	
C5	1.1361 (7)	0.1168 (3)	0.8187 (5)	0.0478 (15)	
C6	1.2298 (7)	0.0529 (4)	0.8537 (5)	0.0583 (17)	
H5	1.2534	0.0389	0.9340	0.070*	
C7	1.2853 (8)	0.0120 (4)	0.7713 (6)	0.0662 (18)	
H7	1.3495	−0.0298	0.7938	0.079*	
C8	1.2449 (8)	0.0333 (4)	0.6505 (6)	0.0608 (17)	
H8	1.2824	0.0039	0.5940	0.073*	
C9	1.1020 (6)	0.1352 (3)	0.6951 (4)	0.0391 (13)	
C10	0.8914 (7)	0.2810 (3)	0.4948 (5)	0.0508 (15)	
H10A	0.9416	0.3268	0.5318	0.061*	
H10B	0.7903	0.2776	0.5152	0.061*	
C11	0.8788 (7)	0.2845 (3)	0.3596 (5)	0.0537 (16)	
H11A	0.8270	0.3315	0.3295	0.064*	
H11B	0.9808	0.2854	0.3406	0.064*	
C12	0.7917 (7)	0.2168 (3)	0.2986 (4)	0.0498 (15)	
H12A	0.6867	0.2192	0.3110	0.060*	
H12B	0.8370	0.1699	0.3353	0.060*	
C13	0.7914 (7)	0.2139 (4)	0.1658 (5)	0.0517 (15)	
C14	0.5817 (7)	0.0439 (4)	0.1021 (5)	0.0523 (15)	
H14	0.5915	0.0389	0.0224	0.063*	
C15	0.4960 (7)	−0.0130 (3)	0.1556 (5)	0.0491 (15)	
C16	0.4348 (7)	−0.0766 (4)	0.0895 (6)	0.0593 (16)	
H16	0.4488	−0.0832	0.0110	0.071*	
C17	0.3533 (8)	−0.1299 (4)	0.1419 (7)	0.075 (2)	
H17	0.3116	−0.1715	0.0960	0.090*	
C18	0.3892 (9)	−0.0646 (5)	0.3151 (7)	0.076 (2)	
H18	0.3749	−0.0600	0.3938	0.091*	
C19	0.4703 (7)	−0.0070 (4)	0.2711 (5)	0.0581 (17)	
H19	0.5068	0.0350	0.3186	0.070*	
C20	0.8106 (7)	0.0064 (3)	0.8035 (5)	0.0442 (14)	
C21	0.8736 (7)	−0.0391 (4)	0.7273 (5)	0.0558 (16)	
H21	0.9340	−0.0807	0.7577	0.067*	
C22	0.8485 (8)	−0.0239 (4)	0.6043 (6)	0.0649 (19)	
H22	0.8948	−0.0546	0.5543	0.078*	
C23	0.7566 (8)	0.0355 (4)	0.5573 (5)	0.0649 (19)	
H23	0.7396	0.0446	0.4752	0.078*	
C24	0.6872 (7)	0.0832 (4)	0.6320 (5)	0.0512 (16)	
C25	0.5906 (8)	0.1436 (4)	0.5884 (6)	0.070 (2)	
H25	0.5701	0.1542	0.5068	0.084*	
C26	0.5250 (8)	0.1879 (4)	0.6658 (6)	0.070 (2)	
H26	0.4589	0.2280	0.6378	0.083*	
C27	0.5617 (8)	0.1702 (4)	0.7890 (6)	0.0663 (18)	
H27	0.5194	0.2010	0.8412	0.080*	
C28	0.7132 (7)	0.0686 (3)	0.7572 (5)	0.0455 (14)	
C29	0.9213 (7)	−0.0686 (3)	0.9732 (5)	0.0523 (15)	
H29A	1.0227	−0.0659	0.9533	0.063*	
H29B	0.8731	−0.1161	0.9414	0.063*	
C30	0.9317 (7)	−0.0652 (3)	1.1079 (5)	0.0472 (14)	
H30A	0.8291	−0.0649	1.1259	0.057*	
H30B	0.9839	−0.1107	1.1441	0.057*	
C31	1.0176 (7)	0.0060 (3)	1.1622 (5)	0.0479 (14)	
H31A	1.1217	0.0044	1.1477	0.057*	
H31B	0.9684	0.0514	1.1232	0.057*	
C32	1.0214 (7)	0.0116 (3)	1.2951 (5)	0.0492 (14)	
C33	1.2327 (7)	0.1813 (4)	1.3530 (5)	0.0517 (15)	
H33	1.2256	0.1860	1.4333	0.062*	
C34	1.3162 (6)	0.2387 (3)	1.2971 (5)	0.0448 (14)	
C35	1.3416 (7)	0.2328 (4)	1.1804 (5)	0.0580 (17)	
H35	1.3053	0.1904	1.1339	0.070*	
C36	1.4204 (8)	0.2895 (4)	1.1339 (6)	0.071 (2)	
H36	1.4347	0.2840	1.0553	0.085*	
C37	1.4515 (8)	0.3568 (4)	1.3043 (6)	0.0665 (19)	
H37	1.4879	0.4002	1.3482	0.080*	
C38	1.3756 (7)	0.3035 (4)	1.3600 (6)	0.0603 (17)	
H38	1.3640	0.3107	1.4389	0.072*	

### Atomic displacement parameters (Å^2^)

**Table:**

	*U*^11^	*U*^22^	*U*^33^	*U*^12^	*U*^13^	*U*^23^
N1	0.053 (3)	0.049 (3)	0.043 (3)	0.012 (2)	0.012 (2)	0.000 (2)
N2	0.068 (4)	0.063 (4)	0.042 (3)	−0.004 (3)	0.017 (3)	0.005 (2)
N3	0.052 (3)	0.061 (3)	0.041 (3)	0.000 (3)	0.010 (2)	0.001 (3)
N4	0.084 (5)	0.072 (5)	0.091 (5)	−0.008 (4)	0.020 (4)	0.010 (4)
N5	0.051 (3)	0.052 (3)	0.045 (3)	−0.006 (3)	0.005 (2)	0.000 (2)
N6	0.070 (4)	0.050 (3)	0.036 (3)	−0.004 (3)	0.019 (3)	0.003 (2)
N7	0.056 (3)	0.042 (3)	0.045 (3)	0.000 (2)	0.012 (2)	0.004 (2)
N8	0.069 (4)	0.059 (4)	0.075 (4)	−0.009 (3)	0.016 (3)	0.007 (3)
O1	0.067 (3)	0.046 (2)	0.036 (2)	0.013 (2)	0.0091 (18)	0.0019 (17)
O2	0.110 (4)	0.072 (3)	0.055 (3)	−0.020 (3)	0.031 (3)	0.010 (2)
O3	0.066 (3)	0.054 (3)	0.033 (2)	0.010 (2)	0.0065 (18)	0.0019 (17)
O4	0.111 (4)	0.060 (3)	0.048 (2)	−0.026 (3)	0.032 (3)	0.002 (2)
O5	0.059 (6)	0.055 (5)	0.102 (7)	0.002 (4)	0.018 (5)	−0.020 (5)
C1	0.057 (4)	0.037 (3)	0.035 (3)	−0.005 (3)	0.009 (3)	−0.001 (2)
C2	0.063 (4)	0.041 (3)	0.047 (4)	−0.002 (3)	0.017 (3)	−0.011 (3)
C3	0.073 (4)	0.055 (4)	0.043 (3)	−0.006 (4)	0.021 (3)	−0.013 (3)
C4	0.068 (4)	0.069 (5)	0.031 (3)	−0.019 (4)	0.002 (3)	−0.004 (3)
C5	0.049 (4)	0.050 (4)	0.041 (3)	0.000 (3)	0.000 (3)	−0.001 (3)
C6	0.057 (4)	0.074 (5)	0.039 (4)	0.003 (4)	−0.003 (3)	0.009 (3)
C7	0.064 (4)	0.070 (5)	0.062 (4)	0.019 (4)	0.004 (3)	0.014 (4)
C8	0.069 (4)	0.058 (4)	0.058 (4)	0.016 (4)	0.018 (3)	0.002 (3)
C9	0.046 (3)	0.037 (3)	0.034 (3)	−0.001 (3)	0.006 (2)	0.000 (2)
C10	0.061 (4)	0.039 (3)	0.050 (4)	0.003 (3)	0.004 (3)	−0.002 (3)
C11	0.065 (4)	0.049 (4)	0.047 (4)	0.001 (3)	0.010 (3)	0.006 (3)
C12	0.062 (4)	0.051 (4)	0.036 (3)	0.004 (3)	0.007 (3)	0.008 (3)
C13	0.057 (4)	0.054 (4)	0.044 (3)	0.002 (3)	0.011 (3)	0.007 (3)
C14	0.060 (4)	0.059 (4)	0.039 (3)	0.010 (3)	0.013 (3)	0.002 (3)
C15	0.046 (4)	0.049 (4)	0.049 (4)	0.003 (3)	0.003 (3)	0.004 (3)
C16	0.068 (4)	0.048 (4)	0.061 (4)	0.009 (3)	0.009 (3)	−0.006 (3)
C17	0.078 (5)	0.052 (5)	0.093 (6)	−0.003 (4)	0.007 (4)	−0.006 (4)
C18	0.075 (5)	0.084 (6)	0.069 (5)	0.006 (5)	0.017 (4)	0.006 (4)
C19	0.068 (4)	0.060 (4)	0.046 (4)	−0.003 (4)	0.009 (3)	0.003 (3)
C20	0.051 (3)	0.048 (3)	0.033 (3)	−0.006 (3)	0.003 (3)	−0.004 (3)
C21	0.063 (4)	0.064 (4)	0.041 (3)	−0.003 (3)	0.010 (3)	−0.011 (3)
C22	0.072 (5)	0.077 (5)	0.050 (4)	−0.019 (4)	0.023 (3)	−0.019 (4)
C23	0.072 (4)	0.087 (5)	0.036 (3)	−0.023 (4)	0.010 (3)	−0.004 (4)
C24	0.053 (4)	0.062 (4)	0.037 (3)	−0.021 (3)	0.005 (3)	0.004 (3)
C25	0.071 (5)	0.080 (5)	0.053 (4)	−0.017 (4)	−0.004 (4)	0.015 (4)
C26	0.065 (5)	0.065 (5)	0.070 (5)	−0.006 (4)	−0.009 (4)	0.020 (4)
C27	0.067 (5)	0.060 (4)	0.071 (5)	0.007 (4)	0.013 (4)	0.006 (4)
C28	0.046 (3)	0.055 (4)	0.035 (3)	−0.013 (3)	0.008 (3)	−0.006 (3)
C29	0.067 (4)	0.040 (3)	0.048 (3)	0.001 (3)	0.005 (3)	−0.002 (3)
C30	0.058 (4)	0.036 (3)	0.045 (3)	0.003 (3)	0.004 (3)	0.010 (3)
C31	0.058 (4)	0.044 (3)	0.042 (3)	−0.004 (3)	0.010 (3)	0.007 (3)
C32	0.064 (4)	0.045 (3)	0.040 (3)	−0.001 (3)	0.013 (3)	0.002 (3)
C33	0.054 (4)	0.055 (4)	0.045 (4)	−0.001 (3)	0.005 (3)	−0.001 (3)
C34	0.047 (3)	0.039 (3)	0.049 (4)	0.005 (3)	0.011 (3)	0.000 (3)
C35	0.060 (4)	0.056 (4)	0.057 (4)	−0.011 (3)	0.007 (3)	−0.009 (3)
C36	0.079 (5)	0.080 (5)	0.057 (4)	−0.018 (4)	0.023 (4)	0.004 (4)
C37	0.071 (5)	0.050 (4)	0.075 (5)	−0.005 (4)	0.005 (4)	−0.008 (4)
C38	0.062 (4)	0.060 (4)	0.059 (4)	−0.002 (4)	0.012 (3)	0.000 (3)

### Geometric parameters (Å, °)

**Table:**

N1—C8	1.314 (7)	C12—H12A	0.9700
N1—C9	1.361 (6)	C12—H12B	0.9700
N2—C13	1.373 (8)	C14—C15	1.451 (8)
N2—N3	1.368 (6)	C14—H14	0.9300
N2—H2	0.8600	C15—C19	1.378 (8)
N3—C14	1.277 (7)	C15—C16	1.391 (8)
N4—C18	1.314 (9)	C16—C17	1.378 (9)
N4—C17	1.335 (9)	C16—H16	0.9300
N5—C27	1.306 (8)	C17—H17	0.9300
N5—C28	1.381 (7)	C18—C19	1.382 (9)
N6—C32	1.349 (7)	C18—H18	0.9300
N6—N7	1.371 (6)	C19—H19	0.9300
N6—H6	0.8600	C20—C21	1.368 (7)
N7—C33	1.278 (7)	C20—C28	1.425 (8)
N8—C37	1.317 (8)	C21—C22	1.398 (8)
N8—C36	1.339 (9)	C21—H21	0.9300
O1—C1	1.359 (6)	C22—C23	1.364 (9)
O1—C10	1.438 (6)	C22—H22	0.9300
O2—C13	1.224 (7)	C23—C24	1.411 (8)
O3—C20	1.354 (6)	C23—H23	0.9300
O3—C29	1.445 (7)	C24—C25	1.389 (9)
O4—C32	1.212 (7)	C24—C28	1.421 (7)
O5—H39	0.8500	C25—C26	1.377 (9)
O5—H40	0.8499	C25—H25	0.9300
C1—C2	1.373 (7)	C26—C27	1.410 (9)
C1—C9	1.429 (7)	C26—H26	0.9300
C2—C3	1.400 (8)	C27—H27	0.9300
C2—H1	0.9300	C29—C30	1.518 (7)
C3—C4	1.342 (9)	C29—H29A	0.9700
C3—H3	0.9300	C29—H29B	0.9700
C4—C5	1.414 (8)	C30—C31	1.524 (8)
C4—H4	0.9300	C30—H30A	0.9700
C5—C6	1.402 (8)	C30—H30B	0.9700
C5—C9	1.417 (7)	C31—C32	1.507 (8)
C6—C7	1.343 (9)	C31—H31A	0.9700
C6—H5	0.9300	C31—H31B	0.9700
C7—C8	1.402 (9)	C33—C34	1.460 (8)
C7—H7	0.9300	C33—H33	0.9300
C8—H8	0.9300	C34—C38	1.386 (8)
C10—C11	1.521 (7)	C34—C35	1.389 (8)
C10—H10A	0.9700	C35—C36	1.373 (8)
C10—H10B	0.9700	C35—H35	0.9300
C11—C12	1.506 (8)	C36—H36	0.9300
C11—H11A	0.9700	C37—C38	1.370 (9)
C11—H11B	0.9700	C37—H37	0.9300
C12—C13	1.509 (7)	C38—H38	0.9300
			
C8—N1—C9	118.0 (5)	C16—C17—H17	118.0
C13—N2—N3	119.3 (5)	N4—C18—C19	125.2 (7)
C13—N2—H2	120.4	N4—C18—H18	117.4
N3—N2—H2	120.4	C19—C18—H18	117.4
C14—N3—N2	116.1 (5)	C15—C19—C18	118.6 (6)
C18—N4—C17	115.7 (6)	C15—C19—H19	120.7
C27—N5—C28	117.0 (5)	C18—C19—H19	120.7
C32—N6—N7	121.2 (4)	O3—C20—C21	125.0 (5)
C32—N6—H6	119.4	O3—C20—C28	115.0 (5)
N7—N6—H6	119.4	C21—C20—C28	119.9 (5)
C33—N7—N6	116.2 (5)	C20—C21—C22	121.0 (6)
C37—N8—C36	114.6 (6)	C20—C21—H21	119.5
C1—O1—C10	117.2 (4)	C22—C21—H21	119.5
C20—O3—C29	116.7 (4)	C23—C22—C21	120.5 (6)
H39—O5—H40	106.4	C23—C22—H22	119.8
O1—C1—C2	124.6 (5)	C21—C22—H22	119.8
O1—C1—C9	115.8 (4)	C22—C23—C24	120.6 (6)
C2—C1—C9	119.5 (5)	C22—C23—H23	119.7
C1—C2—C3	120.2 (6)	C24—C23—H23	119.7
C1—C2—H1	119.9	C25—C24—C23	122.8 (6)
C3—C2—H1	119.9	C25—C24—C28	117.8 (6)
C4—C3—C2	121.4 (5)	C23—C24—C28	119.3 (6)
C4—C3—H3	119.3	C24—C25—C26	120.2 (6)
C2—C3—H3	119.3	C24—C25—H25	119.9
C3—C4—C5	121.1 (6)	C26—C25—H25	119.9
C3—C4—H4	119.5	C25—C26—C27	117.7 (7)
C5—C4—H4	119.5	C25—C26—H26	121.2
C6—C5—C9	117.8 (5)	C27—C26—H26	121.2
C6—C5—C4	123.8 (6)	N5—C27—C26	125.2 (7)
C9—C5—C4	118.4 (5)	N5—C27—H27	117.4
C7—C6—C5	120.0 (6)	C26—C27—H27	117.4
C7—C6—H5	120.0	N5—C28—C24	122.2 (6)
C5—C6—H5	120.0	N5—C28—C20	119.1 (5)
C6—C7—C8	118.8 (6)	C24—C28—C20	118.7 (5)
C6—C7—H7	120.6	O3—C29—C30	106.6 (4)
C8—C7—H7	120.6	O3—C29—H29A	110.4
N1—C8—C7	123.9 (6)	C30—C29—H29A	110.4
N1—C8—H8	118.1	O3—C29—H29B	110.4
C7—C8—H8	118.1	C30—C29—H29B	110.4
N1—C9—C5	121.5 (5)	H29A—C29—H29B	108.6
N1—C9—C1	119.2 (5)	C31—C30—C29	111.8 (5)
C5—C9—C1	119.4 (5)	C31—C30—H30A	109.2
O1—C10—C11	107.2 (4)	C29—C30—H30A	109.2
O1—C10—H10A	110.3	C31—C30—H30B	109.2
C11—C10—H10A	110.3	C29—C30—H30B	109.2
O1—C10—H10B	110.3	H30A—C30—H30B	107.9
C11—C10—H10B	110.3	C32—C31—C30	112.0 (5)
H10A—C10—H10B	108.5	C32—C31—H31A	109.2
C12—C11—C10	111.7 (5)	C30—C31—H31A	109.2
C12—C11—H11A	109.3	C32—C31—H31B	109.2
C10—C11—H11A	109.3	C30—C31—H31B	109.2
C12—C11—H11B	109.3	H31A—C31—H31B	107.9
C10—C11—H11B	109.3	O4—C32—N6	119.7 (5)
H11A—C11—H11B	107.9	O4—C32—C31	123.8 (6)
C11—C12—C13	113.1 (5)	N6—C32—C31	116.5 (5)
C11—C12—H12A	109.0	N7—C33—C34	120.0 (5)
C13—C12—H12A	109.0	N7—C33—H33	120.0
C11—C12—H12B	109.0	C34—C33—H33	120.0
C13—C12—H12B	109.0	C38—C34—C35	116.0 (5)
H12A—C12—H12B	107.8	C38—C34—C33	120.6 (5)
O2—C13—N2	120.0 (5)	C35—C34—C33	123.4 (5)
O2—C13—C12	123.5 (6)	C36—C35—C34	119.9 (6)
N2—C13—C12	116.5 (5)	C36—C35—H35	120.0
N3—C14—C15	120.5 (5)	C34—C35—H35	120.0
N3—C14—H14	119.8	N8—C36—C35	124.3 (6)
C15—C14—H14	119.8	N8—C36—H36	117.9
C19—C15—C16	117.3 (6)	C35—C36—H36	117.9
C19—C15—C14	122.7 (5)	N8—C37—C38	126.1 (6)
C16—C15—C14	120.0 (5)	N8—C37—H37	117.0
C17—C16—C15	119.1 (6)	C38—C37—H37	117.0
C17—C16—H16	120.5	C37—C38—C34	119.0 (6)
C15—C16—H16	120.5	C37—C38—H38	120.5
N4—C17—C16	124.0 (7)	C34—C38—H38	120.5
N4—C17—H17	118.0		
			
C13—N2—N3—C14	−178.6 (5)	N4—C18—C19—C15	−1.3 (11)
C32—N6—N7—C33	179.8 (5)	C29—O3—C20—C21	−1.9 (8)
C10—O1—C1—C2	4.5 (8)	C29—O3—C20—C28	176.6 (5)
C10—O1—C1—C9	−176.7 (5)	O3—C20—C21—C22	−179.2 (6)
O1—C1—C2—C3	179.5 (5)	C28—C20—C21—C22	2.3 (8)
C9—C1—C2—C3	0.7 (8)	C20—C21—C22—C23	−1.9 (9)
C1—C2—C3—C4	0.4 (9)	C21—C22—C23—C24	0.9 (9)
C2—C3—C4—C5	−0.8 (9)	C22—C23—C24—C25	−178.9 (6)
C3—C4—C5—C6	−178.7 (6)	C22—C23—C24—C28	−0.3 (9)
C3—C4—C5—C9	0.1 (9)	C23—C24—C25—C26	179.4 (6)
C9—C5—C6—C7	0.8 (9)	C28—C24—C25—C26	0.9 (9)
C4—C5—C6—C7	179.6 (6)	C24—C25—C26—C27	1.0 (10)
C5—C6—C7—C8	−1.5 (10)	C28—N5—C27—C26	0.3 (9)
C9—N1—C8—C7	−0.1 (9)	C25—C26—C27—N5	−1.7 (11)
C6—C7—C8—N1	1.3 (11)	C27—N5—C28—C24	1.7 (8)
C8—N1—C9—C5	−0.7 (8)	C27—N5—C28—C20	−179.9 (6)
C8—N1—C9—C1	179.8 (5)	C25—C24—C28—N5	−2.3 (8)
C6—C5—C9—N1	0.4 (8)	C23—C24—C28—N5	179.1 (5)
C4—C5—C9—N1	−178.5 (5)	C25—C24—C28—C20	179.3 (5)
C6—C5—C9—C1	179.9 (5)	C23—C24—C28—C20	0.7 (8)
C4—C5—C9—C1	1.0 (8)	O3—C20—C28—N5	1.2 (7)
O1—C1—C9—N1	−0.8 (7)	C21—C20—C28—N5	179.9 (5)
C2—C1—C9—N1	178.1 (5)	O3—C20—C28—C24	179.6 (5)
O1—C1—C9—C5	179.7 (5)	C21—C20—C28—C24	−1.7 (8)
C2—C1—C9—C5	−1.4 (8)	C20—O3—C29—C30	177.7 (5)
C1—O1—C10—C11	179.5 (5)	O3—C29—C30—C31	−64.3 (6)
O1—C10—C11—C12	63.8 (6)	C29—C30—C31—C32	177.2 (5)
C10—C11—C12—C13	−173.9 (5)	N7—N6—C32—O4	176.0 (5)
N3—N2—C13—O2	−176.0 (5)	N7—N6—C32—C31	−5.4 (8)
N3—N2—C13—C12	4.5 (8)	C30—C31—C32—O4	−0.4 (8)
C11—C12—C13—O2	−1.8 (8)	C30—C31—C32—N6	−179.0 (5)
C11—C12—C13—N2	177.7 (5)	N6—N7—C33—C34	180.0 (5)
N2—N3—C14—C15	−178.6 (5)	N7—C33—C34—C38	175.0 (6)
N3—C14—C15—C19	3.4 (9)	N7—C33—C34—C35	−5.0 (9)
N3—C14—C15—C16	−177.0 (6)	C38—C34—C35—C36	−0.5 (9)
C19—C15—C16—C17	−0.2 (9)	C33—C34—C35—C36	179.5 (6)
C14—C15—C16—C17	−179.8 (5)	C37—N8—C36—C35	−0.9 (11)
C18—N4—C17—C16	1.3 (11)	C34—C35—C36—N8	0.5 (11)
C15—C16—C17—N4	−1.2 (10)	C36—N8—C37—C38	1.4 (10)
C17—N4—C18—C19	0.0 (11)	N8—C37—C38—C34	−1.5 (10)
C16—C15—C19—C18	1.4 (9)	C35—C34—C38—C37	0.9 (9)
C14—C15—C19—C18	−179.1 (6)	C33—C34—C38—C37	−179.1 (5)

### Hydrogen-bond geometry (Å, °)

**Table:**

*D*—H···*A*	*D*—H	H···*A*	*D*···*A*	*D*—H···*A*
C31—H31B···O3	0.97	2.55	2.908 (7)	102
C22—H22···O4^i^	0.93	2.51	3.224 (7)	134
C12—H12B···O1	0.97	2.52	2.900 (7)	103
C7—H7···N8^ii^	0.93	2.55	3.466 (9)	167
C3—H3···O2^iii^	0.93	2.52	3.353 (7)	150
C2—H1···N4^iv^	0.93	2.55	3.390 (9)	150
O5—H39···O4^v^	0.85	2.17	2.964 (10)	156
N6—H6···N1^iii^	0.86	2.10	2.934 (6)	163
N2—H2···N5^i^	0.86	2.25	3.077 (7)	161

Symmetry codes: (i) *x*, *y*, *z*−1; (ii) −*x*+3, *y*−1/2, −*z*+2; (iii) *x*, *y*, *z*+1; (iv) −*x*+1, *y*+1/2, −*z*+1; (v) −*x*+1, *y*+1/2, −*z*+2.
